# Differences in spatial niche of terrestrial mammals when facing extreme snowfall: the case in east Asian forests

**DOI:** 10.1186/s12983-024-00522-6

**Published:** 2024-02-01

**Authors:** Hiroto Enari, Haruka S. Enari, Tatsuhito Sekiguchi, Motohisa Tanaka, Sohsuke Suzuki

**Affiliations:** Faculty of Agriculture, Yamagata University, 1-23 Wakabamachi, Tsuruoka, Yamagata 997-8555 Japan

**Keywords:** Climate extreme, Locomotion cost, Niche shift, Niche width, Snow, Temperate forest

## Abstract

**Background:**

Recent climate changes have produced extreme climate events. This study focused on extreme snowfall and intended to discuss the vulnerability of temperate mammals against it through interspecies comparisons of spatial niches in northern Japan. We constructed niche models for seven non-hibernating species through wide-scaled snow tracking on skis, whose total survey length was 1144 km.

**Results:**

We detected a low correlation (*r*_*s*_ < 0.4) between most pairs of species niches, indicating that most species possessed different overwintering tactics. A morphological advantage in locomotion cost on snow did not always expand niche breadth. In contrast, a spatial niche could respond to (1) drastic landscape change by a diminishing understory due to snow, possibly leading to changes in predator-prey interactions, and (2) the mass of cold air, affecting thermoregulatory cost and food accessibility. When extraordinary snowfall occurred, the nonarboreal species with larger body sizes could niche shift, whereas the smaller-sized or semi-arboreal mammals did not. In addition, compared to omnivores, herbivores were prone to severe restriction of niche breadth due to a reduction in food accessibility under extreme climates.

**Conclusions:**

Dietary habits and body size could determine the redundancy of niche width, which may govern robustness/vulnerability to extreme snowfall events.

**Supplementary Information:**

The online version contains supplementary material available at 10.1186/s12983-024-00522-6.

## Background

There is growing evidence that recent global warming has been affecting the distribution of various fauna and flora in the world [1, 2]. Although climate warming affects all seasons, the rise in ambient temperature is projected to be more pronounced in winter, particularly at mid and high latitudes [3]. Low winter temperature produces an increase in the thermoregulatory cost of terrestrial mammals, resulting in energetic bottlenecks that could serve as a direct constrain on geographic distribution, especially for cold-sensitive species [4, 5]. Given this, the current winter warming may create an opportunity to expand the occupancy areas of habitat generalists, as symbolized by the term “tropicalization,” which means the transformation of temperate ecosystems by poleward-moving tropical organisms [6].

When shifting the attention from mean climate on a decadal scale to interannual climate variability, we realize another fact. The recent climate should also be characterized by the expanding fluctuation of meteorological conditions, or the rise in frequency and intensity of extreme climate events such as heat waves, drought, and deluges [7], including cold waves [8, 9]. The biological processes of populations or assemblages are highly sensitive to such extreme climatic events, which are far more than just changes in climatic means [6]. In this respect, the recent climate change is not always a preferable opportunity for improving the fitness of cold-sensitive species, but could rather, in some cases, result in further narrowing the energetic bottleneck observed in winter due to unprecedented cold events [6, 10].

This study focuses on massive snowfall caused by cold waves, one of the possible population bottleneck factors through the physically and physiologically constraining behaviors of terrestrial mammals [10]. To our knowledge, the influence of sudden heavy snowfall on wintering behaviors to keep the fitness of non-hibernating terrestrial mammals has remained poorly understood. As for some specific species groups distributed in North America, however, there have been relatively sufficient studies that may contribute to generating hypotheses worth examining regarding the influence of abnormal snowfall, namely on large-sized ungulates [11–13] and meso- and large-sized canids [14–16].

Those studies commonly indicate that massive snow increases the locomotion cost of mammals. Some metabolic studies targeted at ungulates have demonstrated that locomotion cost rises from two- to six-fold higher when the surface of snow cover reaches the chest or belly height [11, 13]. It should be noted here that the physical property of snow (i.e., snow compaction) related to “how deep legs sink into the snow,” rather than just accumulated ground snow depth, influences the net cost of locomotion [11]. In addition, differences in morphological traits among animals (such as leg length and an area of foot soles affecting leg mobility and snow flotation, respectively) could be a species-specific factor in determining locomotion cost [11, 17]. Similarly, snow also increases the cost of foraging for herbivorous mammals, especially for grazers, which forage plants on the ground [18]. This is because deeper and harder snow requires more energy expenditure to dig for forage [19, 20], and also more strictly constrains visual and olfactory cues to find food [11]. Hence, snow-vulnerable mammals may adopt risk-averse behaviors for saving activity costs through the following tactics: (1) altering habitat selections by being attracted to land with less snow such as evergreen coniferous bottomland, as shown in the cases of some ungulates [13, 21] and in the cases of primates [22, 23]; (2) changing activity budget for minimizing daily travel distance, especially by placing less significance on searching for preferable food [23, 24]; and (3) constraining travel speed by refraining from using “galloping,” which requires large energy consumption per unit distance especially on snow [11].

One of the world’s heaviest snowfall is observed in the northern region of the Japanese archipelago along the coast of the Japan Sea due to its unique geographical location, affected by extremely low ambient temperature and moist atmosphere available from the Siberian Cold Front and warm ocean currents, respectively [25]. This region is located in temperate latitudes, which are the typical regions prone to extreme climate events in winter due to the recent shift of the polar vortex [8, 9]; in fact, severe cold spells have been more frequently observed in recent years [26, 27]. Under such destabilizing winter climates, various terrestrial mammals—including ungulates, carnivores, lagomorphs, and primates—have been widely distributed [28]. We then proposed the following two hypotheses to deepen our understanding regarding the influence of behavioral constraints caused by heavy snow on the spatial niche of temperate-adapted mammals.

### H1

Temperate mammals that are more vulnerable to snow in terms of morphological traits show narrower niche breadth under snowfall conditions, resulting in a reduction in available habitats in a geographical space or actual environmental conditions accessible on the field.

### H2

Extreme snowfall events compel snow-vulnerable mammals to further narrow niche breadth or lead to niche shift (i.e., a change in the centroid of “niche envelope,” or the mean niche position in a multidimensional environmental space [29]).

To test these hypotheses, we constructed ecological niche models for various non-hibernating terrestrial mammals based on repeated and widespread surveys by tracking footprints on snow in northern Japan. By verifying discrepancies in spatial niches among different species and detecting niche variability among different climate conditions, this paper discusses the potential vulnerability of temperate mammals against extreme snowfall as a future concern caused by climate change.

## Materials and methods

### Study area and target species

We set the study area within heavy snowfall regions of northern Japan, which belongs to the cool-temperate climatic zone (range, 37° N–41° N and 139° E–141° E; Additional file 1: Fig. S1). In a normal year, heavy snowfall is observed from December to March and maximum snow depth reaches around 1 m in low-lying regions including residential districts, and 2–5 m in montane regions (Additional file 1: Fig. S1). Similar forest covers are distributed in this area with altitudes below 800 m above sea level; namely, deciduous broadleaf forests (main canopy trees are composed of beech [*Fagus crenata*] and oak [*Quercus crispula* or *Q. serrata*)]) and evergreen conifers (mostly artificial plantations of cedar [*Cryptomeria japonica*]) are mixed in a mosaic-like manner [30].

As middle- and large-sized terrestrial mammals (body weight of adults, > 1 kg), two ungulates (wild boar [*Sus scrofa*] and Japanese serow [*Capricornis crispus*]), five carnivores (Asian black bear [*Ursus thibetanus*], Japanese badger [*Meles anakuma*], red fox [*Vulpes vulpes*], raccoon dog [*Nyctereutes procyonoides*], and Japanese marten [*Martes melampus*]), one lagomorph (Japanese hare [*Lepus brachyurus*]), and one primate (Japanese macaque [*Macaca fuscata*]) are commonly observed as indigenous species in the study area [28]. Among them, we treated only non-hibernating species (i.e., seven species, excluding bears and badgers) as the target species of this study.

There is a morphological method to estimate the snow tolerance of mammals, known as the morphological index (MI), which is calculated as follows: MI = MCH + [100 − (MFL/10)], where MCH is mean chest height (cm) and MFL is mean foot loading (g/cm^2^) [17]. Higher MI values represent greater snow-tolerant ability in terms of morphological plasticity. When calculating MI values for the above seven mammals, the results (in descending order) were 127 for macaques, 125 for foxes, 113 for both hares and raccoon dogs, 112 for martens, 110 for serows, and 92 for boars (as for the data source and calculation process, see Additional File 1: Table S1).

### Data collection

To build ecological niche models for verifying the current hypotheses, we adopted the tracks of target mammals left on the snow surface as credible evidence of species presence. Given that each target mammal possesses readily identifiable differences in footprint morphology and/or gait pattern [31, 32], we safely distinguished the species only by their tracks. We set 55 survey transects in low-mountain forests with mosaic landscapes below 800 m above sea level within the Towada, Asahi, and Oguni regions, located in northern Japan (Additional File 1: Fig. S1 as for the properties of land use, see Table 1). In most areas in these regions, maximum snowfall with > 2 m in depth was normally observed and the recent distributions of target mammals were less influenced by human disturbances such as deforestation and hunting. The total length of the transects was 21 km in Towada, 180 km in Asahi, and 111 km in Oguni. We followed the transects on skis during the daytime when snowfall, which obliterated mammal tracks from the snow surface, had not been observed for > 24 h before each survey. Then, we recorded a geolocation of distinct tracks only when those intersected transects. We conducted this survey once a mid-winter (i.e., mostly from February to March, when the deepest snowpack was observed) from 2016 to 2020 in Towada, from 2014 to 2019 in Asahi, and from 2015 to 2019 in Oguni. Although we could not follow all the transects every year because of limitations due to weather conditions, the total length of the transects that we explored reached 1144 km. The mean completion percentage of transects surveyed each year was 90% in Towada, 52% in Asahi, and 87% in Oguni.

**Table 1:** Explanatory variables with a 50-m grid resolution for modeling ecological niches of terrestrial mammals in snowfall regions of northern Japan

Category	Explanatory variables (unit)	Description	Range	Sources
(a) Regulation factors	Maximum snow depth (cm)	Mean values observed between 1991 to 2020	37–378	A
	Solar radiation (KWh/m^2^)	Incoming solar radiation accumulated during the survey months (February and March)	60–199	B
(b) Disturbance factors	Distance to dwelling land (m)	Distance to areas occupied by artificial architecture	0–1994	C
	Snowfield (250 m^2^)	Area without any vegetation cover available during winters (grassland, shrubland, paddies, or cropland)	0–1	C
	West-facing slope (250 m^2^)	Slope land facing the bearing angle between 225° and 315°, affected by the winter monsoon (i.e., powerful and cold wind)	0–1	D
(c) Resources	Evergreen conifer (250 m^2^)	Area covered by evergreen conifer plantation, providing refuge from snowfall and cold wind	0–1	C
	Deciduous broadleaf forest (250 m^2^)	Area covered by deciduous trees, providing plant food for herbivores	0–1	C
(d) Others	Elevation (m)	Mean elevation	16–782	D
	Slope angle (°)	Mean slope angle	0–58	D

A: “Climate mesh data,” published by Digital National Land Information, Ministry of Land, Infrastructure and Transport, Japan

B: We calculated the values from the digital elevation model (composed of 10-m grid cells), published by Geospatial Information Authority of Japan, by using “solar radiation toolset” in ArcGIS 10.8.1

C: Land-use data as of 2018–2020 based on “High-resolution land use, land cover map ver. 21.11 (composed of 10-m grid cells),” published by Japan Aerospace Exploration Agency

D: Digital elevation model (as above)

### Interspecies comparisons of spatial niche

To test H1 regarding the interspecific differences in spatial niche observed under snowfall conditions, we built ecological niche models of each target species by using the following three approaches with the package ENMTools 1.1.1 [33] under the *R* platform v.4.2.3 [34]: a regression-based approach “generalized additive model, GAM” [35], an entropy-based approach “MAXENT” [36], and the bagging approach “random forest, RFO” [37].

To obtain a reasonable interpretation of niche models, the determination of spatial resolution assigned for modeling should require the consideration of spatial uncertainty of species presence data [38]. Considering present snow tracking could provide high-accuracy coordinate values of species presence, we set spatial grid resolution to “50 m × 50 m” for the current modeling. This scale corresponded to the highest resolution available in the dataset for explanatory variables (described later) and is also an empirically known grid size that could provide good performance to explain daily habitat use under heavy snow conditions [39, 40]. We used all the track data recorded on transects as presence data (i.e., objective variable) after performing “spatial filtering” [41] by removing duplicated tracks within each grid cell for all the targeted species. Consequently, we obtained presence data with less spatial autocorrelation, composed of 139 boar tracks, 235 macaque tracks, 181 fox tracks, 523 marten tracks, 1026 hare tracks, 693 raccoon dog tracks, and 566 serow tracks. We allocated 80% of those presence points to model training and the remaining 20% to model testing for each target species.

Regardless of which niche modeling approach is adopted, the spatial extent of the reference area, where species pseudo-absence or background data are selected, is crucial to enhance the model performance [42, 43]. Considering that the present transects are scattered throughout northern Japan, we restricted the reference area to a 100-m buffer area for every transect, which consisted of 22,518 grid cells. Within the reference area, we randomly extracted 5,000 grid cells as pseudo-absence data, accounting for 22.2% of the reference area. More commonly, the spatial niche of species is defined only in a geographical space (i.e., available combinations of environmental conditions in a reference area). However, if the conditions within a reference area are composed of a biased subset of potential environments, the measure of spatial niche may also be biased [44]. Hence, we defined the spatial niche also in an environmental space generated by “Latin hypercube sampling,” indicating that there are no discrete units and predetermined limits to the maximum/minimum values of each predictor variable [44]. For this processing, we used the *R* package ENMTools 1.1.1 [33] and selected 10,000 random points from an environmental space as pseudo-absence or background data.

Afterward, we prepared explanatory variables within the reference area. Environmental predictors defining species spatial niche are generally divided into three categories: (1) regulation factors, which affect species metabolic activities; (2) disturbance factors, or natural/artificial processes constraining species distribution; and (3) resources, which are integral species-specific components of habitats [38]. Given this, we prepared nine explanatory variables for the current models (Table 1), which were common environmental predictors to possibly explain the occurrence of each species based on previous studies [39, 40, 45–47]. We confirmed that no high-variance inflation factors (i.e., > 3) existed between all possible combinations of the variables, indicating a low level of multicollinearity [48].

To build niche models, we used all the explanatory variables without any interactions. When conducting GAM, we assigned a binomial family (link function = logit) with a penalized regression spine with smoothing parameters determined by restricted maximum likelihood. As for MAXENT, we used the regularization multiplier, which determines the degree of model generality by mitigating model complexity and overfitting [36], with 1 as the default value. When performing RFO, we used the out-of-bag observations in each tree to measure the prediction errors. We determined the discrimination accuracy by using the area under the receiver operating curves (AUC). The value of AUC ranges from 0.5 (no predictive performance) to 1.0 (perfect prediction). According to the recommendation by Swets JA [49], we decided that AUC values > 0.8 and < 0.6 indicated “good” and “fail,” respectively. Using the AUC for the test data (20% of presence points) in the environmental space, we adopted a final model among the three different modeling approaches for each species. We evaluated the contribution of each explanatory variable to the final model by using the permutation-based method according to the AUC metric [50]. In this method, the variable importance is calculated by iteratively omitting each variable when building a model and measuring the reduction in the AUC value. For this, we set the number of Monte Carlo replications at 10.

We then measured the correlation of predicted models in the environmental space for each pair of species to identify niche overlap among species [44]. We used Spearman’s rank correlation coefficient (*r*_*s*_) as a metric, which could emphasize differences in the physiological response to each predictor [33]. To identify the interspecies variations in niche breadth under snowfall conditions, we calculated Levin’s *B2* in the environmental space [44].

$${x}_{i}=\frac{{y}_{i}}{\sum _{i}^{n}{y}_{i}}$$

$$B2=\frac{1}{n-1}\left(\frac{1}{{\sum }_{i}^{n}{x}_{i}^{2}}-1\right)$$

where *x*_*i*_ is the standardized suitability value in the *i*th environmental condition (expressed as a combination of environmental variables within a reference area), *y*_*i*_ is the suitability value in the *i*th environmental condition, and *n* is the total number of environmental conditions. For this calculation under environmental space, we set chunk size (the combination number of environmental variables to be processed at a time) and tolerance (a parameter set for computation precision) at default values, i.e., 100,000 and 0.0001. *B2* values range from 0 to 1 and the larger value indicates wider niche breadth or habitat generalists. Finally, we compared the habitats of each target species actually observed in the geographical space by using an index, total habitat unit (THU), defined as “habitat quality × habitat quantity” [51]. Here we measured THU as a total sum of suitability values observed in each grid cell within the reference area.

### Niche variability among different winter climates

To test H2 regarding the niche variability of each species among different winter climates, we compared spatial niches predicted under normal and extraordinary heavy snowfall years. We observed extreme climate events with heavy snowfall during the winters of 2015 and 2018 in the Oguni region, when the snow accumulation reached 221 and 242 cm deep, respectively, even at the lowest altitude areas (i.e., 140 m above sea level) in this region. This snow depth was twice as much as that observed in normal snowfall years (111 cm in 2016 and 125 cm in 2017). We then assigned two-year pooled data of tracks observed each in the normal and heavy snowfall years, which were extracted from the same data set as above, and built ecological niche models in this region using the GAM approach. Consequently, we employed 34 tracks for boars, 177 for hares, 49 for macaques, 72 for martens, 125 for raccoon dogs, and 76 for serows during the heavy snowfall years, and 47 for boars, 288 for hares, 27 for macaques, 153 for martens, 220 for raccoon dogs, and 109 for serows during the normal snowfall years. Boars and macaques are typical gregarious mammals; therefore, we counted their tracks in units of groups to avoid increasing spatial autocorrelation. There were limited fox tracks that we observed and therefore, we abandoned building models for this species. To keep the minimum sample size for building niche models, i.e., 20–50 presence points [52], we allocated all the presence data to model training.

All procedures of the model construction (including the selections of spatial resolution, explanatory variables, reference area, and GAM setting) and model evaluation were the same as those in the above section, except for the number of pseudo absence data, which were randomly selected from geographic space, i.e., 1,000 grid cells, accounting for 34.2% of the reference area in this analysis. Based on these models, we conducted pairwise niche identity tests in the environmental space [53] using ENMtools 1.1.1 [33] to see whether the obtained correlation coefficient (*r*_*s*_) of paired niche models predicted under the normal and heavy snowfall conditions significantly differed from a null distribution, assuming no niche differentiation (*α* = 0.05). We here created the null distribution for each species by repeatedly calculating *r*_*s*_ between two datasets, which were randomly generated from the pooled species presence data under both conditions. For this, we set the number of Monte Carlo replicates at 100.

We then identified niche variability or niche conservatism under fluctuating winter climates by two metrics: niche breadth change and niche shift. We calculated niche breadth change as differences in Levin’s *B2* in the environmental space which were measured under normal and heavy snowfall conditions. For this calculation, we used the same parameter setting as those in the above section. As for the niche shift, we evaluated a change in the centroid of niche envelope defined under the multi-dimensional environmental space, i.e., inertia ratio [29]. For this, we identified the observed value of each explanatory variable (composed of the standardized value ranging from 0 to 1) that posed the highest suitability values in each model and measured the total sum of variations in those observed values between models predicted under the normal and heavy snowfall years. A lower inertia ratio indicates fewer niche shifts, or a more strict niche conservatism [54].

## Results

### Interspecies comparisons of spatial niche

**Fig. 1:**
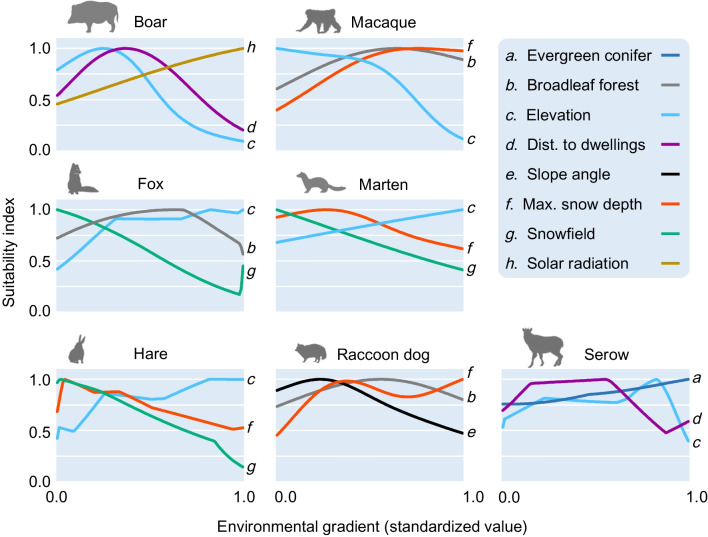
Response curves for environmental variables with the top 3 variable importance, provided by the best habitat model of each mammal species, in the snowfall regions of northern Japan. The contribution ratios of these three variables to the total value of variable importance extracted from all the variables was 88.7% for boars, 53.2% for foxes, 70.8% for hares, 80.8% for macaques, 69.4% for martens, 73.6% for raccoon dogs, and 55.1% for serows

Responding to the AUC-based model selections, we adopted GAM to predict the spatial niche for boars (AUC value for the test data in the environmental space = 0.89), macaques (0.83), martens (0.91), and raccoon dogs (0.80) and MAXENT for foxes (0.87), hares (0.90), and serows (0.75). Given that most of the AUC values were > 0.80, those final models could successfully possess favorable predictive performance. As shown in the top 3 permutation-based variable importance of obtained niche models for each species (Fig. 1; for detailed variable importance, see Additional file 1: Fig. S2), “elevation” was the most common influential predictor (six out of seven species), followed by “maximum snow depth” (four out of seven species). Whereas foxes, martens, hares, and serows were commonly good at high-elevation areas, boars and macaques avoided those areas. Increasing snow depth positively influenced macaques and raccoon dogs but adversely affected martens and hares. The occurrence of a “snowfield” (an area without any vegetation covers) commonly decreased the habitat suitability of the smaller-sized mammals (foxes, hares, and martens).

**Fig. 2:**
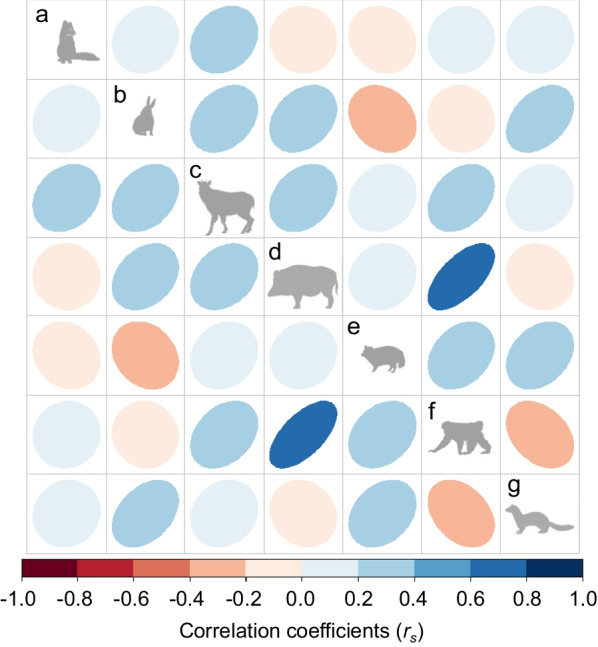
Correlation matrix of habitat suitability values provided by ecological niche models for the seven mammal species **a** red fox, **b** Japanese hare, **c** Japanese serow, **d** wild boar, **e** raccoon dog, **f** Japanese macaque, and **g** Japanese marten in the environmental space

**Fig. 3:**
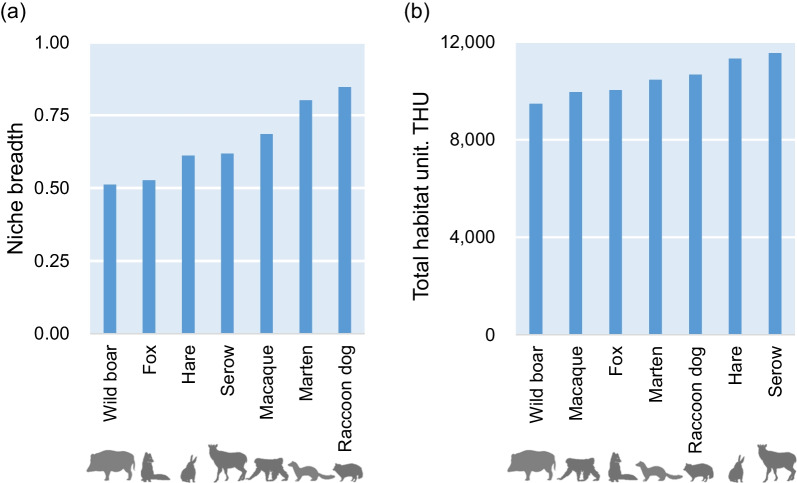
Niche breadth (Levin’s *B2*) in the environmental space and total habitat unit in the geographic space (i.e., total of habitat suitability values observed at each grid within the reference area) targeted for terrestrial mammals in snowfall regions of northern Japan

As shown in the correlation matrix of predicted models in the environmental space (Fig. 2), most pairs of species niche were not correlated with each other (*r*_*s*_ < 0.4), except for the pairs “marten × hare” and “macaque × boar.” The predicted niche breadth in the environmental space showed that, whereas the mesocarnivores (raccoon dogs and martens) were habitat generalists, boars and foxes were specialists (Fig. 3a). The THU confirmed in the geographic space indicated that boars possessed the most limited habitats under snowfall conditions (Fig. 3b), while hares and serows ensured large suitable habitats there. The morphological index (MI; Additional  file 1: Table S1) was not significantly correlated both with niche breadth and THU (*p* = 0.59, *r*_*s*_ = 0.25 for niche breadth; *p* = 0.78, *r*_*s*_ = − 0.12 for THU).

### Niche variability among different winter climates

Most of the current GAM-based niche models, constructed under different snow conditions, exhibited favorable performance. Specifically, the AUC values of the models during heavy and normal snowfall in the environmental space were 0.80 and 0.86 for boars; 0.93 and 0.68 for hares; 0.79 and 0.88 for martens; 0.86 and 0.75 for raccoon dogs; 0.75 and 0.82 for serows; and 0.88 and 0.77 for macaques, respectively. Therefore, we decided to use all of them in subsequent analyses. The niche identity test to compare niche models under normal and heavy snowfall conditions rejected the null hypothesis (niche equivalency) for boars (*p* = 0.04, empirical *r*_*s*_ = 0.04, null distribution *r*_*s*_ = 0.56 ± 0.18 SD), hares (*p* = 0.01, empirical *r*_*s*_ = 0.20, null distribution *r*_*s*_ = 0.79 ± 0.12), raccoon dogs (*p* = 0.01, empirical *r*_*s*_ = 0.28, null distribution *r*_*s*_ = 0.85 ± 0.11), and serows (*p* = 0.02, empirical *r*_*s*_ = − 0.13, null distribution *r*_*s*_ = 0.15 ± 0.20). In contrast, the identity tests for martens (*p* = 0.32, empirical *r*_*s*_ = 0.31, null distribution *r*_*s*_ = 0.37 ± 0.17), and macaques (*p* = 0.14, empirical *r*_*s*_ = 0.17, null distribution *r*_*s*_ = 0.51 ± 0.27) did not reject the null hypothesis, indicating no significant difference in niche models under different snowfall conditions.

**Fig. 4:**
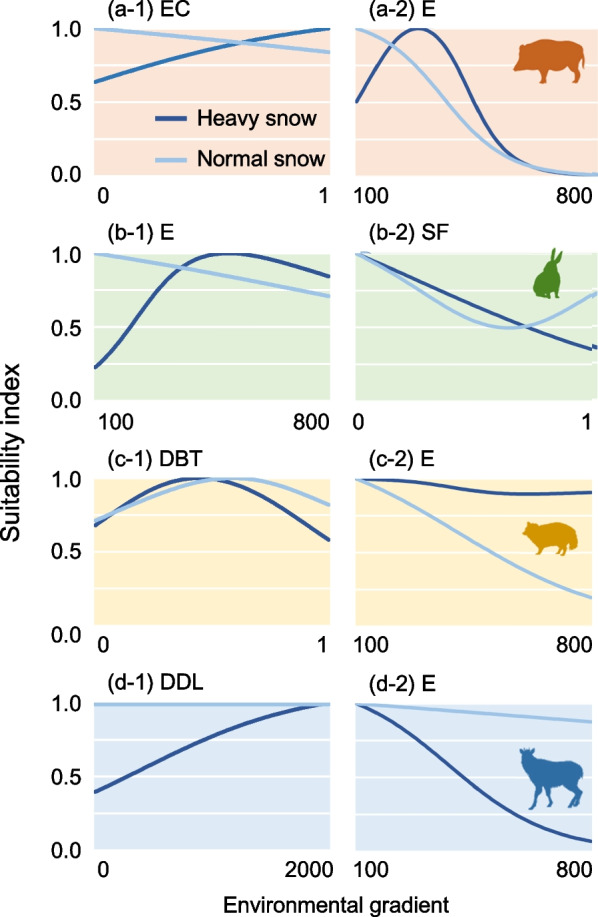
Comparing the ecological niches observed during the normal and heavy snowfall years for each mammal species (**a** boar, **b** hare, **c** raccoon dog, and **d** serow) in the Oguni region, northern Japan. Each panel shows response curves for the respective explanatory variables with the top 2 variable importance, determined by the total of “variable importance” of each variable in the normal and heavy snowfall years’ models. We omitted comparing the response curves for the species without significant differences in ecological niche, judged by the niche identity test (i.e., martens and macaques). EC: evergreen conifer [250 m^2^], E: elevation [m], SF: snowfield [250 m^2^], DBT: deciduous broadleaf tree [250 m^2^], and DDL: distance to dwelling land [m]

For the four mammals with significant differences in niche models, we demonstrated the response curves of explanatory variables with the top 2 variable importance (Fig. 4;  for detailed variable importance, see Additional file 1: Fig. S3). Based on this, boars increased their frequency of use for evergreen conifers under heavy snowfall conditions. Hares avoided the low-land areas under heavy snowfall conditions. Whereas raccoon dogs rarely used the high-land areas under normal snowfall conditions, serows avoided those areas under heavy snowfall conditions. In addition, serows did not frequently occupy the areas bordering on human settlements under heavy snowfall conditions.

**Fig. 5:**
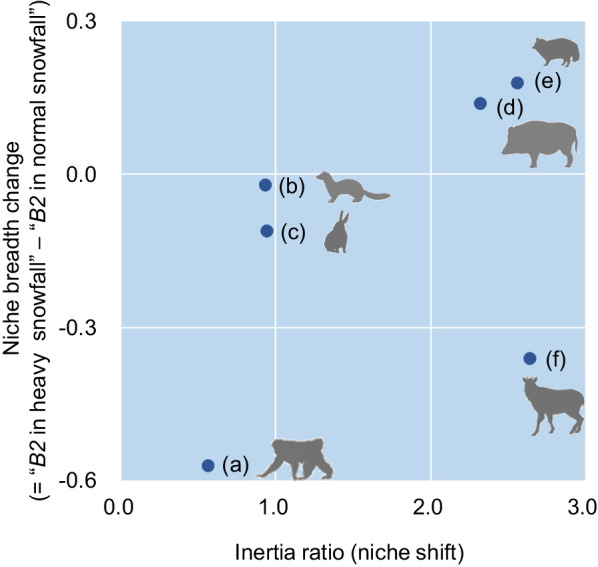
Scatter plots of inertia ratios (i.e., the amount of niche shift) versus the changes in amount of niche breadth (Levin’s *B2* in environmental space) between the normal and heavy snowfall years in northern Japan: **a** macaque, **b** marten, **c** hare, **d** boar, **e** raccoon dog, and **f** serow. A lower inertia ratio indicates less variation in the centroid of the niche envelope (the mean position in *n*-dimensional environmental space) under different snowfall conditions (i.e., more strict niche conservatism)

We created the scatter plots composed of two axes, i.e., niche breadth change and inertia ratio (Fig. 5). As for niche breadth change, the typical omnivores (boars and raccoon dogs) showed positive values, meaning niche breadth tends to increase under heavy snowfall conditions. In contrast, the herbivores (macaques, serows, and hares) exhibited negative values, indicating a narrower niche breadth observed in extremely heavy snowfall conditions. When focusing on inertia ratio, whereas macaques, martens, and hares exhibited niche conservatism, large-sized mammals (boars and serows) and raccoon dogs exhibited large niche shifts.

## Discussion

### Interspecies differences in spatial niches

The wide-scale spatial niche, estimated in the three typical snowfall regions of northern Japan, presented the low correlation values between the most pairs of temperate mammal species (Fig. 2), even among the same guild, i.e., mesocarnivores with opportunistic omnivorousness [28]. This dissimilarity may indicate that each mammal species possesses species-specific tactics of habitat uses to overwinter under snowfall conditions, as discussed below.

Among the target mammal species, wild boars with the most unsuitable morphology to snow according to the MI value (Additional  file  1: Table S1)  distinctly maintained a passive attitude to endure snowfall conditions, as evidenced by the narrowest niche breadth and THU (Fig. 3). This result could be compatible with earlier observations in high-latitude forests that a > 50-cm-deep snowpack is a critical factor to restrict the population fitness of boars [55–57]. Few correlations between the MI values and niche breadths (or THUs), however, could lead to the conclusion that most mammal species, excluding boars, are far less likely to follow the prediction of H1, i.e., under massive snowfall conditions, the morphological advantage in locomotion cost on snow largely determines the available habitats of species. Typical rebuttal evidence has been observed for macaques with the highest MI values among the target species (Additional  file 1: Table S1). Despite the greater robustness against snowfall detected in the model prediction (Fig. 1), the niche breadth of macaques remained moderate, and their available habitats were the second narrowest after those of boars (Fig. 3). In contrast, although serows exhibited the second lowest MI values after boars, they had the largest suitable habitats in northern Japan regions with deep snow (Fig. 3b).

Key factors behind such rebuttals could be gleaned from the multifaceted impacts of snow on non-hibernating mammals’ habitats that do not involve locomotion costs. Massive snow accumulation drastically alters, albeit seasonally, landscape structure by diminishing understory vegetation. This drastic change (emerging “snowscape”) has been regarded as a possible factor that causes a variation in predator-prey dynamics by increasing the area of snowfields without coverts for prey animals against predators [58, 59]. As predicted by those earlier arguments, snowfields with high predation risk became a glaring constraint of habitat uses for hares (Fig. 1). Along with this, it should be considered that martens and foxes, serving as typical predators for hares [60, 61], were forced to avoid snowfields with fewer prey animals (Fig. 1).

A mass of cold air itself, bringing in massive snowfall, may be another factor to disprove the H1 prediction for some mammal species. Altitudinal gradient (directly linked to ambient temperature) and/or the amount of solar radiation became reducing factors of niche breadth and THU for macaques and boars (Fig. 1), the only pair of species to share the closely similar spatial niche (Fig. 2). Incidentally, low ambient temperature, especially with temperatures lower than − 5 °C, could directly increase the thermoregulatory cost for macaques, which could largely constrain their activity budget including feeding behaviors [62]. Such severe behavioral constraint may be due to the evolutionary perspective that the macaques originated as a relative species (*Macaca robustus*) that adapted to the warm-temperate climate of continental China [63, 64]. Conversely, the thermoregulatory cost observed in northern Japan is unlikely to restrict the spatial niche of boars. This is because the animals can also inhabit higher-latitude regions close to the Arctic Circle [65] by possessing greater cold tolerance, allowing them to survive under the average winter temperature below − 30 °C [57]. However, the Asian winter monsoon (i.e., strong cold winds) originating in low ambient temperatures leads to hard-packed snow, possibly strongly constraining food accessibility for boars, which employ a rooting behavior when foraging by digging snow [55, 56].

It should also be noted that the availability of preferable habitats was not always expanded for the species with a wider niche breadth in the environmental space, or habitat generalists (Fig. 3). Specifically, although hares and serows had intermediate niche breadth, those animals held the largest habitats in present-day northern Japan. Such an inconsistent trend may be caused by evolutionarily optimizing the spatial niche of those species by preferentially fitting for the geographic space with the regional-specific environmental conditions, characterized by vegetation, terrain, and climates, strongly disturbed/influenced by the Asian winter monsoon. In fact, the plasticity specialized for snowfall has been found only in those two species; namely, while hares inhabiting northern Japan have obtained morphological plasticity for heavy snowfall (i.e., larger hind foot length [66] and whiter pelage during winters [67]), serows have been considered habitat and dietary specialists only for cool-temperate forests with snowfall [28, 68]. In contrast, omnivorous mesocarnivores (i.e., raccoon dogs and martens) with the largest niche breadth posed constricted adaptability to the present-day geographic space (Fig. 3). However, this gap, or the redundancy of niche breadth toward existing environmental conditions, may be a superior tolerance against fluctuating environments caused by future climate warming.

### Spatial niche variations observed under extraordinary snowfall

As evidenced in the above section, wild boars could be considered the most vulnerable species to snow in terms of morphology, niche breadth, and habitat availability. Boars in snowy regions originally occupied only lower-elevation forests with less snowfall (Fig. 1), which means that altitudinal migration for avoiding additional snowfall is not a feasible option. Hence, the animals endured climate extremes by occupying the areas with evergreen conifer cover (Fig. 4). Similar tactics to endure heavy snowfall have been well-known in other large ungulates (i.e., cervids) and dense conifer has been considered an essential resource serving as an emergency shelter from extreme snowfall [13, 21, 69]. Such evacuation behaviors observed for boars possibly resulted in niche shifts caused by extreme snowfall events (Fig. 5).

Along with boars, the nonarboreal species with larger body sizes, mostly exhibiting relatively large MI values (Additonal File 1: Table S1), were prone to showing larger niche shifts when extraordinary snowfall came (Fig. 5) by adopting different behavioral tactics. Serows, originally regarded as habitat generalists in terms of elevation, drastically changed their niche centroid under extraordinary snowfall, mainly by avoiding higher-elevation forests with deeper snow (Fig. 4d). As for raccoon dogs, an opposite trend triggered the niche shift, which posed the altitudinal preference only during the normal snowfall years (Fig. 4c). Extraordinary snowfall also provided massive snowpacks in lower-elevation forests, which attained a depth of > 2 m (see Material and methods). Therefore, it is possible that the cost-saving advantage of altitudinal migrations (from high to low elevations), especially to dig snow for forage (mainly carcasses), was hardly available even in lower-elevation forests during the heavy snowfall years. Meanwhile, we detected “niche conservatism” for smaller-sized or semi-arboreal mammals (i.e., hares, martens, and macaques), which employed tactics by taking little initiatives to shift the centroid of the niche envelope even under the drastic changes of winter climate.

Thus, the above findings could support the part of the H2 prediction regarding niche shift (i.e., extraordinary snowfall events compel “niche shift” or “further restriction of niche breadth” for snow-vulnerable species). Contrary to this, the dietary habits of species, rather than potential snow vulnerability, could determine the change in niche breadth caused by extraordinary snowfall, which indicates the disconfirmation of the H2 prediction regarding niche breadth. The change in niche breadth predicted by the present models demonstrated that the typical omnivores, including necrophagy especially during winters (i.e., raccoon dogs and boars; [70, 71] ), reflected a trend toward habitat generalists by using wider environmental conditions when extraordinary snowfall came (Fig. 5). Such behavioral changes may be explainable in terms of not only a remarkable tolerance to cold, including in arctic/subarctic climates, observed for those mammals (boars [57]; raccoon dog [72]), but also the improvement of food accessibility. Specifically, extreme snowfall events often result in increasing the mortality rate of temperate large-sized mammals [73–75], which increases the opportunities for detecting food (i.e., carcasses) for opportunistic scavenger mammals in response to search efforts (a similar example shown in foxes [*Vulpes lagopus*] of the Arctic area [59]). Incidentally, a recent study indicates that, even if the visual cue to search carcasses is constrained by massive snowfall, opportunistic scavengers can effectively detect them by relying solely on olfactory cues and > 90% of the carcasses buried in snow can be completely consumed during winter [70].

As opposed to omnivores, the niche breadth of herbivores—i.e., macaques, serows, and hares, regarded as bark/bud eaters during winters [39]—was strongly restricted during the extraordinary snowfall. The rationale for such a narrower niche breadth could be relatively simple; extreme snowfall massively covered understory plants, which are their main dietary resources [23, 74, 76]. However, attention should be paid to the fact that such an influence of extraordinary snowfall was limited not only to nonarboreal herbivores but to semi-arboreal ones, i.e., macaques. A recent study may answer these observations, demonstrating that macaques overwinter under extreme snowfall events by drastically switching their diets from understory plants to overstory trees (which are abundant and easily accessible resources) for saving locomotion costs to search for food [23]. Hence, such drastic risk averse behaviors could result in a large restriction of niche breadth for macaques, even when compared to nonarboreal species.

### Potential vulnerability of temperate mammals against extreme snowfall events

This study also generated a simple but new understanding that every species occupied the regions with much deeper snowpack than their chest height, a possible tipping point to sharply increase energy expenditure demands [11, 13]. For example, boars, regarded as a typical snow-vulnerable species, were commonly observed in forests with > 2-m snow accumulation, which is three to four times deeper than the threshold limit of their range that previous studies indicated [57, 75]. Considering that the populations of every species, including boars, have been constantly maintained or expanding, rather than decreasing, even in the regions with such snow conditions as of now [77–79], the current wintering tactics that the present niche models revealed could hardly lead to decreasing their population fitness under the current geographic space.

Under further growing uncertainty concerning future climate change [80], the above argument may not always conduce to an optimistic forecast that the projected ecological risks of future climate extremes, called “pulse events” [7], are negligible for temperate mammals. Considering the severe restriction of niche breadth and niche conservatism observed under such pulse events, let alone boars, even the species excelling at the previous snowfall conditions, such as hares and serows, may not avoid those adverse impacts on their fitness if the change in the available conditions of the geographic space exceeds the redundancy or plasticity of their spatial niche. Although we could not discuss the actual impacts of pulse events on the population fitness of temperate mammals, this study provides an incentive for the ecological community to enrich our understanding regarding possible species turnover (examples in flora [81] and in avifauna [82]) caused by not only changing the climatic mean but also such climate pulse events.

### Supplementary Information

**Additional file 1.** **Fig. S1** shows geographical placement of survey transects and maximum snow depth in eastern Japan. **Fig. S2** shows permutation-based variable importance for the best niche model of each mammal species in the snowfall regions of northern Japan. **Fig. S3.** shows mean and SD of variable importance in the predicted ecological niche of each mammal species under different snowfall conditions in northern Japan. **Table S1** shows relative degree of morphological plasticity to cope with the snow for adults of each mammal species in northern Japan.

## Data Availability

All original data used in this research have been deposited in Figshare (https://doi.org/10.6084/m9.figshare.24188430.v1). All environmental dataset that we used for the current analyses are publicly available (sources provided in Table 1).
