# Supplementary material for: Differences in spatial niche of terrestrial mammals when facing extreme snowfall: the case in east Asian forests
Source: Front Zool. 2024 Feb 1;21:3. doi: 10.1186/s12983-024-00522-6 (PMC10832220; doi:10.1186/s12983-024-00522-6)
Supplement: Supplementary file 1 — Additional file 1. Fig. S1 shows geographical placement of survey transects and maximum snow depth in eastern Japan. Fig. S2 shows permutation-based variable importance for the best niche model of each mammal species in the snowfall regions of northern Japan. Fig. S3. shows mean and SD of variable importance in the predicted ecological niche of each mammal species under different snowfall conditions in northern Japan. Table S1 shows relative degree of morphological plasticity to cope with the snow for adults of each mammal species in northern Japan. [file 12983_2024_522_MOESM1_ESM.docx]

**Additional file 1** for:

**Differences in spatial niche of terrestrial mammals when facing extreme snowfall:**

**the case in east Asian forests**

Hiroto Enari^1^*, Haruka S. Enari^1^, Tatsuhito Sekiguchi^1^, Motohisa Tanaka^1^, Sohsuke Suzuki^1^

^1^Faculty of Agriculture, Yamagata University, 1-23 Wakabamachi, Tsuruoka, Yamagata, 997-8555 Japan

*Correspondence: enari@tds1.tr.yamagata-u.ac.jp

Oguni

Asahi

Towada

Fig. S1. Geographical placement of survey transects (gray lines in the map) and maximum snow depth (mean values between 1991 and 2020 according to the open data “Digital National Land Information,” provided by Japan Ministry of Land, Infrastructure, Transport and Tourism) in eastern Japan.

Fig. S2. Permutation-based variable importance for the best niche model of each mammal species in the snowfall regions of northern Japan. Bars show SD.

Fig. S3. Mean and SD (shown in bars) of variable importance (EC: evergreen conifer, DBF: deciduous broadleaf forest, E: elevation, DDL: distance to dwelling land, SA: slope angle, MSD: maximum snow depth, SF: snowfield, SR: solar radiation, WS: west-facing slope) in the predicted ecological niche of each mammal species under different snowfall conditions in northern Japan: (a) boar, (b) hare, (c) marten, (d) raccoon dog, (e) serow, and (f) macaque.

Table S1. Relative degree of morphological plasticity to cope with the snow for adults of each mammal species in northern Japan

| Species | A: Chest height (cm) ^a^  [ mean size for adults ] | B: Body weight (g) ^b^  [ mean size for adults ] | C: Area of footprints (cm^2^) ^c^  [ mean size for adults ] | D: Foot loading (g/cm^2^)  [ D = B/C ] | E: Morphological index ^d^  [ E = A + (100 − D/10) ] |
| --- | --- | --- | --- | --- | --- |
| Japanese  macaque | 33 ^e^ | 12,760 | 226 | 57 | 127 |
| Red  fox | 36 ^f^ | 4,700 | 42 | 112 | 125 |
| Japanese  hare | 14 ^g^ | 2,350 | 194 | 12 | 113 |
| Raccoon  dog | 25 ^h^ | 4,130 | 34 | 121 | 113 |
| Japanese  marten | 16 ^i^ | 1,284 | 32 | 40 | 112 |
| Japanese  serow | 74 ^b^ | 37,150 | 58 | 641 | 110 |
| Wild  boar | 75 ^b^ | 75,000 | 90 | 833 | 92 |

^a^ We used the mean withers height when there was no measurement for chest height.

^b^ [1]

^c^ [2]

^d^ The equation is based on that of [3]. The higher value indicates a greater snow-coping ability in light of morphology.

^e^ [4]

^f^ [5]

^g^ [6]

^h^ [7]

^i^ Enari, H. unpublished data

**References**

1. Ohdachi SD, Ishibashi Y, Iwasa MA, Saito T: The wild mammals of Japan: second edition. Kyoto: Shoukadoh; 2015.

2. Komiya T: The handbook of mammals feet types and footprints. Tokyo: Bun-ichi; 2013.

3. Telfer ES, Kelsall JP: Adaptation of some large North American mammals for survival in snow. Ecology. 1984; 65:1828-1834.

4. Takatsuki S, Yamagiwa J: Mammalogy in Japan. Tokyo: Tokyo University Press; 2008.

5. Takeuchi M: Sexual dimorphism and relative growth of body size in the Japanese red fox *Vulpes vulpes japonica.* Mamm Study. 2010; 35:125-131.

6. Funo T, Sekijima T, Abe M: Changes in feeding pattern of Golden Eagle *Aquila chrysaetos* with leafing of deciduous trees. Japanese Journal of Ornithology. 2010; 59:148-160.

7. Mae Y, Nagara K, Miyazaki M, Katsura Y, Enomoto Y, Koga A: Complex intragene deletion leads to oculocutaneous albinism in tanuki (Japanese raccoon dog). Genome. 2020; 63:517-523
